# *In Vitro* Biotransformation of Total Glycosides in Qiwei Baizhu Powder by the Gut Microbiota of Normal and Diarrheal Mice: Novel Insight Into the Biotransformation of Multi-Glycosides by the Gut Microbiota 

**DOI:** 10.3389/fchem.2022.907886

**Published:** 2022-06-20

**Authors:** Guozhen Xie, Rongrong Zhou, Lili Huang, Shuihan Zhang, Zhoujin Tan

**Affiliations:** ^1^ School of Pharmacy, Hunan University of Chinese Medicine, Changsha, China; ^2^ The Affiliated Hospital of Hunan Academy of Chinese Medicine, Changsha, China; ^3^ College of Traditional Chinese Medicine, Hunan University of Chinese Medicine, Changsha, China; ^4^ Institute of Chinese Materia Medica, Hunan Academy of Chinese Medicine, Changsha, China; ^5^ College of Medicine, Hunan University of Chinese Medicine, Changsha, China

**Keywords:** total glycosides, Qiwei Baizhu powder, UHPLC-Q-TOF-MS/MS, *in vitro* metabolism, metabolites

## Abstract

The gut microbiota (GM) is involved in the metabolism of glycosides and is beneficial for enhancing their bioactivity. However, the metabolism of multi-glycosides by the GM under normal and pathological conditions is unclear. In this study, the total glycosides (TG) of the traditional Chinese medicine (TCM) formula Qiwei Baizhu Powder (QWBZP) were extracted to represent a multi-glycoside system. Ultra-high-performance liquid chromatography quadrupole time-of-flight tandem mass spectrometry (UHPLC-Q-TOF-MS/MS) was used to rapidly identify the components and *in vitro* metabolites of QWBZP-TG. The metabolic profiles of QWBZP-TG in the GM of normal and diarrheal mice were also compared. A total of 68 compounds and seven metabolites were identified in the QWBZP-TG and metabolic samples, respectively. Deglycosylation was the main metabolic pathway of *in vitro* multi-glycoside metabolism. Liquiritin apioside, isoliquiritin apioside, liquiritin, protopanaxadiol (PPD)-type, and oleanane (OLE)-type ginsenosides were relatively easy to metabolize by the GM. At first, the deglycosylation capability of the GM of normal mice was superior to that of diarrheal mice, but the deglycosylation capability of diarrheal mice gradually recovered and produced abundant deglycosylation metabolites. In conclusion, deglycosylation metabolites may be the bioactive components of QWBZP. Glycoside-bacteria interaction may be a key mechanism for QWBZP to therapy diarrhea.

## Introduction

Glycosides are widely distributed in herbal medicines and exert a wide range of pharmacological activities, including anti-inflammatory, antimicrobial, antipyretic, antidiarrheal, antidiabetic, and antitumor effects (Bartnik and Facey, 2017). However, the bioavailability of glycosides is extremely poor owing to the presence of a saccharide group. Secondary glycosides and/or aglycones, which are less polar and can be absorbed into the bloodstream, primarily influence the therapeutic direction (Bartnik and Facey, 2017; Xu et al., 2017). The traditional Chinese medicine (TCM) formula comprises multiple Chinese materia medica and is often extracted by water to generate a decoction for use in clinical practice. As polar glycosides occur widely in decoctions, the TCM formula is a natural multi-glycoside system.

Qiwei Baizhu Powder (QWBZP), which is composed of Atractylodis Macrocephalae Rhizoma (15 g), Ginseng Radix et Rhizoma (7.5 g), Poria (15 g), Puerariae Lobatae Radix (15 g), Aucklandiae Radix (6 g), Pogostemonis Herba (15 g), and Glycyrrhizae Radix et Rhizoma (3 g), is an ancient TCM formula for the treatment of diarrhea. It is now widely used for treating gastrointestinal diseases, including irritable bowel syndrome and ulcerative colitis (Peng et al., 2014). We have previously reported that QWBZP improves antibiotic-associated diarrhea by regulating the intestinal microecology and exerting anti-inflammatory effects (Hui et al., 2020; Long et al., 2020). Although many beneficial effects have been reported, the actual bioactive components of QWBZP are still not well understood. Ginsenoside Rb_1_ and Rd have been reported to improve carbachol-induced hyperperistalsis in the small intestine (Hashimoto et al., 2003). Glycyrrhizic acid exerted ameliorative effects on colitis by inhibiting the enterotoxin of *Escherichia coli*, improving the pathological changes of colon tissue, and reducing the expression of pro-inflammatory factors and chemokines (Chen et al., 2009). Puerarin, 3′-hydroxypuerarin, daidzin, and daidzein are the active components of pueraria with antidiarrheal effects (Huang et al., 2010). Consequently, we speculated that glycosides mediate the antidiarrheal effects of QWBZP.

The gut microbiota (GM) plays a pivotal role in drug metabolism. It has been reported that the contribution of the GM to drug metabolism is up to 70%, which is higher than that of the host (Zimmermann et al., 2019a). Accumulating evidence supports that the GM-mediated metabolism is key to the pharmacodynamic activities of glycosides. The gut microbiome encodes an enormous number of glycoside hydrolases that cleave glycosidic bonds within glycosides and produce secondary glycosides and/or aglycones (Koppel et al., 2017; Lin et al., 2019). Liu et al. (2019) and Zhu et al. (2021) compared the metabolic differences of glycosides by the GM under normal and pathological conditions and found specific metabolites, suggesting that glycoside metabolism strongly depends on the number and composition of the GM. Although the *in vitro* and *in vivo* metabolism of single glycosides by the GM has been widely studied, the metabolism of multi-glycosides by the GM under normal and pathological conditions has received little attention owing to the chemical complexity.

In this study, we extracted total glycosides (TG) from a QWBZP decoction (QWBZP-TG) to represent a multi-glycoside system. An ultra-high-performance liquid chromatography combined with quadrupole time-of-flight tandem mass spectrometry (UHPLC-Q-TOF-MS/MS) method was used to identify the components of QWBZP-TG and to illustrate the metabolic profile of QWBZP-TG in the GM of normal and diarrheal mice. The objectives of this study were to 1) identify the mechanism of *in vitro* multi-glycoside metabolism by the GM, 2) clarify the metabolic profiles of QWBZP-TG in the GM, and 3) compare the metabolic differences between healthy and pathological states and consider the mechanism of QWBZP in curing diarrhea.

## Experimental

### Materials and Reagents

Acetonitrile (MS grade), methanol (MS grade), and ammonium formate (≥99.0%) were purchased from Merck (Darmstadt, Germany). Formic acid (HPLC grade) was purchased from Tedia (Fairfield, OH, United States). Ultrapure water was supplied using a Millipore Milli-Q purification system (Bedford, MA, United States). Analytical grade anhydrous ethanol, petroleum ether, ethyl acetate, and n-butanol were purchased from Sinopharm Chemical Reagent Co., Ltd (Shanghai, China). Reference standards for ginsenoside Re (DSTDR001402), ginsenoside Rg_1_ (DSTDR000901), ginsenoside Ro (DSTDR003101), puerarin (DSTDG000202), daidzin (DST200706-019), liquiritin (DSTDG000902), liquiritin apioside (DST211025-139), and liquiritigenin (DSTDG001001) were purchased from Chengdu Desite Biological Technology Co., Ltd (Chengdu, China). Dihydrodaidzein (wkq22032909) was purchased from Sichuan Victory Biological Technology Co., Ltd (Chengdu, China). The anaerobic culture medium (HB8470) was purchased from Hope Bio-Technology Co., Ltd (Qingdao, China). Gentamicin sulfate (01Y07011A2) and cefradine (06200502) were purchased from Yichang Renfu Pharmaceutical Co., Ltd and Jilin Wantong Pharmacy Group Co., respectively.

The Chinese herbal slices of QWBZP, including Atractylodis Macrocephalae Rhizoma (*Atractylodes macrocephala*, 20201014, Hunan, China), Ginseng Radix et Rhizoma (*Panax ginseng*, 20200610, Jilin, China), Poria (*Poria cocos*, 20201109, Hunan, China), Puerariae Lobatae Radix (*Pueraria lobata*, 20201020, Hunan, China), Aucklandiae Radix (*Aucklandia lappa*, 20201011, Yunnan, China), Pogostemonis Herba (*Pogostemon cablin*, 20200816, Guangdong, China), and Glycyrrhizae Radix et Rhizoma (*Glycyrrhiza uralensis*, 20200610, Neimenggu, China), were purchased from The First Hospital of Hunan University of Chinese Medicine and were certified by Qingping Pan, a professor at the School of Pharmacy, Hunan University of Chinese Medicine.

### QWBZP-TG Preparation

QWBZP-TG was prepared according to a previously optimized method (Xie G. Z. et al., 2021). Briefly, Chinese herbal slices of QWBZP were pulverized into powder (60 mesh), and then a 14-fold mass of distilled water was added and decocted twice for 53 min each time. The combined decoctions were filtered using gauze and concentrated below 90°C. Anhydrous ethanol was added when the concentrate was cooled to room temperature, and the final concentration of ethanol, which was subsequently stored overnight at 4°C, was 75%. After 24 h, the ethanol extract was successively degreased thrice with petroleum ether, and then extracted thrice with water-saturated n-butanol. The n-butanol extract was evaporated by decompression and lyophilized (yield: 1.48%).

### Sample Preparation and Standard Solution

Dry QWBZP-TG (30 mg) was dissolved in 2 ml of methanol (15 mg/ml) and centrifuged at 15,000 rpm for 5 min prior to UHPLC-Q-TOF-MS/MS analysis. Another 25 mg of dry QWBZP-TG was dissolved in 5 ml sterile water (5 mg/ml) and filtered using a 0.22 μm pore membrane for the *in vitro* metabolism assay.

A stock solution of the nine reference substances was prepared at a concentration of 200 μg/ml and stored at 4°C until use. Standard working solutions of the mixtures were obtained by diluting the stock solutions to the desired concentrations.

### Animal Experiments

Ten male Kunming (KM) mice (five-week-old, 18–22 g, specific pathogen free [SPF] degree), with license number SCXK (Xiang) 2019–0004, were purchased from Hunan Slaccas Jingda Laboratory Animal Co., Ltd. The mice were acclimated in a controlled room with standard temperature (20–22°C), humidity (50–70%), and light (12:12 light-dark cycle) for 3 days. Standard food and purified water were provided ad libitum. The experiments were approved by the Animal Care and Use Committee of Hunan University of Chinese Medicine (authorization number: LL2020102103).

The mice were randomly divided into control and model groups with five mice in each group. The mice in the model group were administered a mixture of gentamycin sulfate and cefradine (62.5 g/L, 0.35 ml) twice daily until diarrhea symptoms were observed (5 days), whereas the mice in the control group were administered the same volume of sterile water.

### Fecal Sample Preparation

After 5 days of treatment, all mice were euthanized by cervical dislocation, and colonic fecal samples were immediately collected under sterile conditions. Fecal suspensions for the *in vitro* metabolism assay were prepared using fresh feces and cold normal saline (1:4, *W*/*V*) and then centrifuged at 4,000 rpm for 10 min.

### *In Vitro* Metabolism of QWBZP-TG by the GM

The anaerobic culture medium (9.5 g) was dissolved in 1,000 ml of water (60 °C), followed by autoclaving (0.15 MPa, 121°C) for 30 min. The GM incubation solution (GMIS) was composed of fecal suspensions and an anaerobic culture solution (ACS) (1:9, *V*/*V*). The GMIS was shaken and cultured (37°C) for 24 h after blowing nitrogen (N_2_).

Five different control experiments were designed: 1) ACS + QWBZP-TG (Y), 2) control mice-GMIS (N), 3) model mice-GMIS (M), 4) control mice-GMIS + QWBZP-TG (NG), and 5) model mice-GMIS + QWBZP-TG (MG). The reaction mixture was designed as shown in Table 1 and anaerobically incubated at 37 °C for 0, 2, 4, 8, 12, and 24 h after blowing N_2_. Three repetitions were performed at each time point for each group. Each reaction mixture was marked by the group name and time; for instance, Y_0, 2, 4, 8, 12, 24_ represented the Y samples at 0, 2, 4, 8, 12, and 24 h, respectively.

**TABLE 1:** The material composition of each group.

Group	ACS (ml)	Control Mice-GMIS (ml)	Model Mice-GMIS (ml)	QWBZP-TG (μl)
Y	2	—	—	80
N	—	2	—	—
M	—	—	2	—
NG	—	2	—	80
MG	—	—	2	80

“—" means absence of this component.

The biotransformation samples were extracted twice with 2 ml of ethyl acetate and a water-saturated n-butanol mixture (1:1, *V*/*V*). Subsequently, the extracts were homogeneously mixed and dried with N_2_. The remaining residues were redissolved in 2 ml methanol and centrifuged at 15,000 rpm for 5 min before analysis.

### UHPLC-Q-TOF-MS/MS Analysis

All analyses were performed using an Agilent 1290 Infinity Ⅱ liquid chromatography (LC) system (Agilent Technologies, Santa Clara, CA, United States) coupled with an Agilent Technologies 6545 quadrupole time-of-flight mass spectrometer (Q-TOF-MS) equipped with an electrospray ionization (ESI) source.

Chromatographic separation was carried out using an Agilent Eclipse Plus C18 column (2.1 mm × 50 mm, 1.8 μm) with a constant flow rate of 0.4 ml/min at 30 °C. The mobile phase was composed of water containing 5 μmol/ml ammonium formate (A) and acetonitrile (B) in the negative ion mode (ESI^−^) and 0.1% formic acid-water (A) and acetonitrile (B) in the positive ion mode (ESI^+^). Gradient elution was set as follows: 95–90% A for 0–10 min, 90%–70% A for 10–16 min, 70%–30% A for 16–21 min, 30%–20% A for 21–25 min, and 20% A for 25–30 min. The injection volume was 2 μl. The samples were detected at 210 and 270 nm.

Q-TOF-MS was operated in both ESI^+^ and ESI^−^. The operating parameters were as follows: drying gas (N_2_) flow rate, 8 L/min; drying gas temperature, 350 °C; nebulizer, 35 psig; capillary, 3.5 kV; sheath gas temperature, 350 °C; sheath gas flow, 11 L/min; October 1 RF Vpp, 750 V; and fragmentor voltage, 150 V. Full scan mass spectra were acquired in the range of 100–2000 m*/z*. The collision energies were set at 10, 20, and 40 V.

### Data Analysis

LC-MS data analysis was performed using the Mass Hunter Workstation software (Version B.08.00 Qualitative Analysis, Agilent Technologies). Statistical analysis was performed using SPSS 21.0 using unpaired *t*-test and data are presented as the mean ± standard deviation (SD). The differences with *p* < 0.05 were considered statistically significant.

## Results

### Characterization of the Components in QWBZP-TG

**FIGURE 1:**
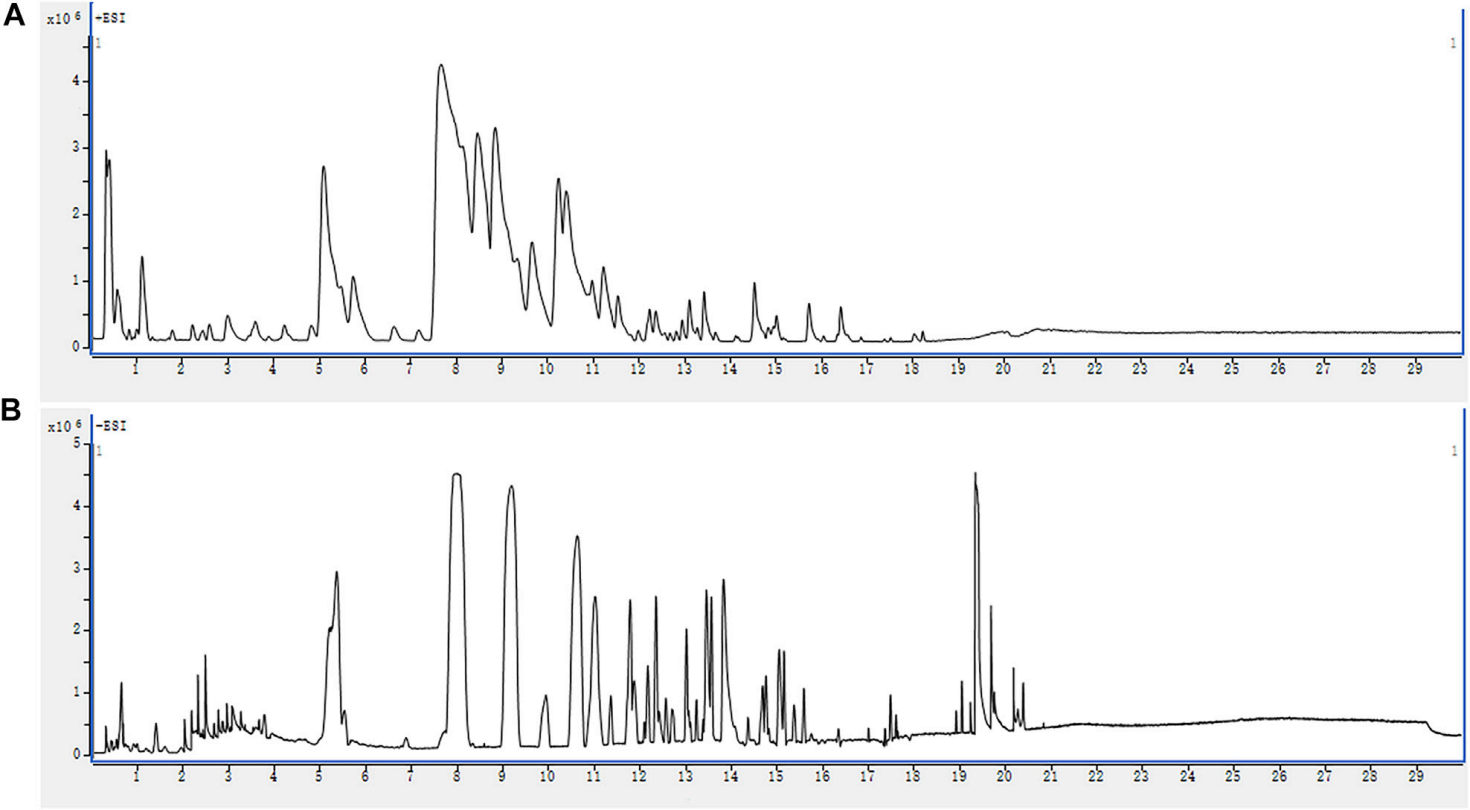
The BPC profiles of QWBZP-TG. **(A)** ESI^+^; **(B)** ESI^−^.

The base peak chromatograms (BPCs) of QWBZP-TG are presented in Figure 1. A total of 68 components were identified in QWBZP-TG on the basis of accurate molecular weight, fragmentation behavior, and by comparison with reference standards or literature information. Details including retention times (*t*_R_), identified names, formulas, experimental and calculated mass *m/z*, errors, and fragment characteristic ions are listed in Table 2.

**TABLE 2:** Identification of chemical constituents of QWBZP-TG.

No.	*t*_R_/min	Identification	Ion Type	Formula	Detected	Expected	Error/ppm	Fragment Ions
1	14.504	ginsenoside Re_1/2/3_	[M + COOH]^-^	C_48_H_82_O_19_	1,007.5372	1,007.5374	0.17	961.5332, 799.4808, 475.3776
2	14.801	ginsenoside Ⅰ/Ⅱ	[M-H]^-^	C_48_H_82_O_20_	977.5340	977.5327	−1.36	931.5295, 799.4875, 637.4370
3	15.083	ginsenoside Rg_1_ ^a^	[M + COOH]^-^	C_42_H_72_O_14_	845.4915	845.4904	−1.29	799.4852, 637.4319, 475.3756
4	15.173	ginsenoside Re^a^	[M + COOH]^-^	C_48_H_82_O_18_	991.5483	991.5483	0.02	945.5426, 783.4830, 475.3764
5	16.243	ginsenoside Ro^a^	[M-H]^-^	C_48_H_76_O_19_	955.4945	955.4967	2.28	793.4372, 731.4375, 613.3730, 523.3793
6	16.659	ginsenoside Rh_1_	[M + COOH]^-^	C_36_H_62_O_9_	683.4379	683.4376	−0.46	637.4394, 475.3792
7	17.083	ginsenoside Rf	[M + COOH]^-^	C_42_H_72_O_14_	845.4903	845.4904	0.13	799.4855, 637.4299, 475.3766
8	17.425	ginsenoside 20(*S*)-Rg_2_	[M + COOH]^-^	C_42_H_72_O_13_	829.4944	829.4955	1.32	783.4904, 637.4315, 619.4218, 475.3795
9	17.480	ginsenoside F_1_	[M + COOH]^-^	C_36_H_62_O_9_	683.4381	683.4376	−0.75	637.4357, 475.3788
10	17.490	ginsenoside 20(*R*)-Rg_2_	[M + COOH]^-^	C_42_H_72_O_13_	829.4919	829.4896	−2.74	783.4909, 637.4320, 475.3779
11	17.493	ginsenoside Rc	[M-H]^-^	C_53_H_90_O_22_	1077.5831	1077.5851	1.85	945.5483, 783.4896, 621.4341
12	17.621	ginsenoside Rb_2_/Rb_3_	[M + COOH]^-^	C_53_H_90_O_22_	1123.5929	1123.5906	−2.07	1077.5813, 945.5400, 621.4305
13	17.906	ginsenoside Rd	[M + COOH]^-^	C_48_H_82_O_18_	991.5468	991.5483	1.53	945.5395, 783.4820, 621.4344, 459.7446
14	18.436	ginsenoside Rg_9_	[M + COOH]^-^	C_42_H_70_O_13_	827.4808	827.4798	−1.15	781.4760, 619.4338
15	18.511	ginsenoside F_2_	[M + COOH]^-^	C_42_H_72_O_13_	829.4953	829.4955	0.24	783.4937, 621.4262
16	18.784	ginsenoside Rg_6_	[M + COOH]^-^	C_42_H_70_O_12_	811.4859	811.4849	−1.19	765.4791, 619.4257
17	18.917	ginsenoside Rk_3_	[M + COOH]^-^	C_36_H_60_O_8_	665.4249	665.4270	3.18	619.4215
18	19.244	ginsenoside 20(*S*)-Rg_3_	[M + COOH]^-^	C_42_H_72_O_13_	829.4948	829.4955	0.84	783.4909, 621.4379, 459.3850
19	19.316	ginsenoside 20(*R*)-Rg_3_	[M + COOH]^-^	C_42_H_72_O_13_	829.4975	829.4955	−2.41	783.4902, 621.4408, 459.3851
20	20.384	ginsenoside F_4_	[M + COOH]^-^	C_42_H_70_O_12_	811.4837	811.4849	1.51	765.4809, 619.4201
21	21.184	ginsenoside Rs_4_	[M + COOH]^-^	C_44_H_72_O_13_	853.4989	853.5014	2.89	807.492, 765.4749
22	16.648	soyasaponin Ba	[M + H]^+^	C_48_H_78_O_19_	959.5168	959.5151	−1.74	779.4533, 599.3917, 441.3692
23	17.250	licorice saponin G_2_	[M-H]^-^	C_42_H_62_O_17_	837.3907	837.3914	0.86	351.0577, 193.0352
24	15.583	glycyrrhizic acid	[M + H]^+^	C_42_H_62_O_16_	823.4077	823.4111	4.09	647.3778, 471.3453, 453.3358
25	18.241	azukisaponin Ⅱ	[M + H]^+^	C_42_H_68_O_14_	797.4657	797.4682	3.12	617.4031, 441.3717, 423.3605
26	14.607	22*β*-acetoxylglycyrrhizic acid	[M-H]^-^	C_44_H_64_O_18_	879.4015	879.4020	0.56	351.0567
27	16.171	licorice saponin A_3_	[M-H]^-^	C_48_H_72_O_21_	983.4509	983.4493	−1.59	821.3961, 351.0669, 193.0324
28	1.914	3′-hydroxypuerarin-4′-*O*-glucoside	[M + H]^+^	C_27_H_30_O_15_	595.1664	595.1657	−1.10	433.1122, 415.1001, 397.0895, 367.0790, 313.0712
29	3.640	puerarin-4′-*O*-*β*-*D*-glucoside	[M + H]^+^	C_27_H_30_O_14_	579.1706	579.1708	0.40	459.1279, 417.1175, 399.1068, 351.0860, 297.0752
30	8.313	puerarin-7-*O*-glucoside	[M-H]^-^	C_27_H_30_O_14_	577.1567	577.1563	−0.73	457.1130, 429.1197, 267.0649
31	4.592	3′-methyoxy-4′-*O*-glucosyl-puerarin	[M + H]^+^	C_28_H_32_O_15_	609.1814	609.1814	−0.01	447.1282, 429.1167, 411.1046, 381.0926, 327.0847
32	12.048	genistein-8-*C*-glucoside	[M-H]^-^	C_21_H_20_O_10_	431.0993	431.0984	−2.15	311.0572, 283.0645
33	5.354	3′-hydroxypuerarin	[M-H]^-^	C_21_H_20_O_10_	431.0993	431.0984	−2.15	311.0566, 283.0622
34	11.732	genistin	[M-H]^-^	C_21_H_19_O_10_	431.0991	431.0984	−1.69	311.0571, 269.0460, 227.0712, 149.0313
35	10.619	6″-*O*-xylosylpuerarin	[M-H]^-^	C_26_H_28_O_13_	547.1471	547.1457	−2.53	295.0616, 267.0694
36	11.981	3′-hydroxypuerarin xyloside	[M-H]^-^	C_26_H_28_O_14_	563.1407	563.1406	−0.13	311.0570, 283.0577
37	7.984	puerarin^a^	[M-H]^-^	C_21_H_20_O_9_	415.1045	415.1035	−2.51	325.0718, 307.0607, 295.0619, 277.0505, 267.0662
38	13.233	3′-methoxypuerarin	[M-H]^-^	C_22_H_22_O_10_	445.1141	445.1140	−0.18	325.0706, 310.0466, 282.0519
39	9.613	mirificin	[M + H]^+^	C_26_H_28_O_13_	549.1601	549.1603	0.31	417.1180, 399.1068, 381.0957, 351.0855, 297.0753
40	12.433	daidzin^a^	[M + H]^+^	C_21_H_20_O_9_	417.1177	417.1180	0.74	255.0653
41	11.651	3′-methoxydaidzin	[M + H]^+^	C_22_H_22_O_10_	447.1281	447.1286	1.06	285.0756, 270.0518, 253.0495, 225.0541
42	12.590	5′-hydroxyl oninin	[M + COOH]^-^	C_22_H_22_O_10_	491.1169	491.1195	5.83	445.1257, 283.0624, 268.0384
43	15.370	4′,6-dimethoxyisoflavone-7-*O*-glucoside	[M-H]^-^	C_23_H_24_O_10_	505.1345	505.1351	1.28	297.0772, 282.0490
44	12.347	genistein-8-*C*-apiosyl (1–6)-glucoside	[M-H]^-^	C_26_H_28_O_14_	563.1409	563.1406	−0.48	311.0558, 283.0638
45	13.560	formonononetin-8-*C*-glucoside-*O*-xyloside	[M-H]^-^	C_27_H_30_O_13_	561.1622	561.1614	−1.49	309.0764, 281.0828
46	13.601	6″-*O*-acetyl daidzin	[M + COOH]^-^	C_23_H_22_O_10_	503.1160	503.1136	−4.18	457.1157, 253.0510, 295.0612, 252.0427
47	14.968	ononin	[M + H]^+^	C_22_H_22_O_9_	431.1330	431.1337	1.53	311.0907, 269.0809, 254.0570, 213.0906
48	5.521	daidzein-4′,7-*O*-glucoside	[M + COOH]^-^	C_29_H_32_O_18_	623.1638	623.1653	2.54	415.1038, 253.0514
49	13.419	4′-*O*-methoxypuerarin	[M-H]^-^	C_22_H_22_O_9_	429.1193	429.1191	−0.45	309.0771, 281.0826, 209.0601
50	15.033	3′-methoxypuerarin-6″-*O*-*β*-Apionoside	[M-H]^-^	C_27_H_30_O_14_	577.1566	577.1563	−0.55	325.0713, 297.0765, 282.0539
51	13.059	apigenin-7-*O*-*β*- *d* -glucopyranoside	[M + COOH]^-^	C_21_H_20_O_10_	477.1053	477.1038	−3.36	431.0988, 269.0456
52	12.010	liquiritin^a^	[M-H]^-^	C_21_H_22_O_9_	417.1170	417.1191	3.60	255.0661, 135.0079, 119.0494
53	12.434	liquiritin apioside^a^	[M-H]^-^	C_26_H_30_O_13_	549.1614	549.1614	−0.06	255.0659, 135.0084
54	15.091	isoliquiritin	[M-H]^-^	C_21_H_22_O_9_	417.1184	417.1191	1.69	255.0662, 135.0086, 119.0480
55	12.708	isoliquiritin apioside or isomer	[M-H]^-^	C_26_H_30_O_13_	549.1616	549.1614	−0.43	255.0667, 119.0494
56	13.455	acteoside	[M-H]^-^	C_29_H_35_O_15_	623.1962	623.1981	3.11	461.1624, 315.1230, 297.0949, 161.0244
57	13.836	isoacteoside	[M-H]^-^	C_29_H_35_O_15_	623.1969	623.1981	1.99	461.1637, 315.5615, 179.0335, 161.0216
58	14.553	leucosceptoside A	[M-H]^-^	C_30_H_37_O_15_	637.2138	637.2138	−0.01	461.1624, 315.1055, 175.0400, 135.0435
59	12.565	pueroside A	[M-H]^-^	C_29_H_34_O_14_	605.1885	605.1876	−1.52	297.0770, 253.0874
60	7.724	genistein	[M-H]^-^	C_15_H_10_O_5_	269.0467	269.0455	−4.27	225.0702, 133.0395
61	12.387	glycitein	[M + H]^+^	C_16_H_12_O_5_	285.0758	285.0757	−0.18	270.0522, 242.0566, 213.0551
62	14.754	formonononetin	[M-H]^-^	C_16_H_12_O_4_	267.0651	267.0663	4.41	252.0430, 224.0040, 167.2342
63	11.724	daidzein	[M-H]^-^	C_15_H_10_O_4_	253.0509	253.0506	−1.05	223.0399, 195.0445, 133.0290
64	15.559	3′-methoxydaidzein	[M + H]^+^	C_16_H_12_O_5_	285.0747	285.0757	3.70	270.0518, 242.0585, 225.0543, 213.0529, 152.0090
65	18.852	irisolidone	[M + H]^+^	C_17_H_14_O_6_	315.0860	315.0863	1.00	300.0628, 272.0661
66	11.584	calycosin	[M + H]^+^	C_16_H_12_O_5_	285.0756	285.0757	0.53	270.0520, 253.0491, 225.0544, 214.0612, 137.0227
67	13.062	apigenin	[M-H]^-^	C_15_H_10_O_5_	269.0469	269.0455	−5.01	225.0569, 197.0600
68	16.613	naringenin	[M-H]^-^	C_15_H_12_O_5_	271.0609	271.0612	1.09	151.0025, 119.0502, 107.0140

a Compared with reference standards.

**FIGURE 2:**
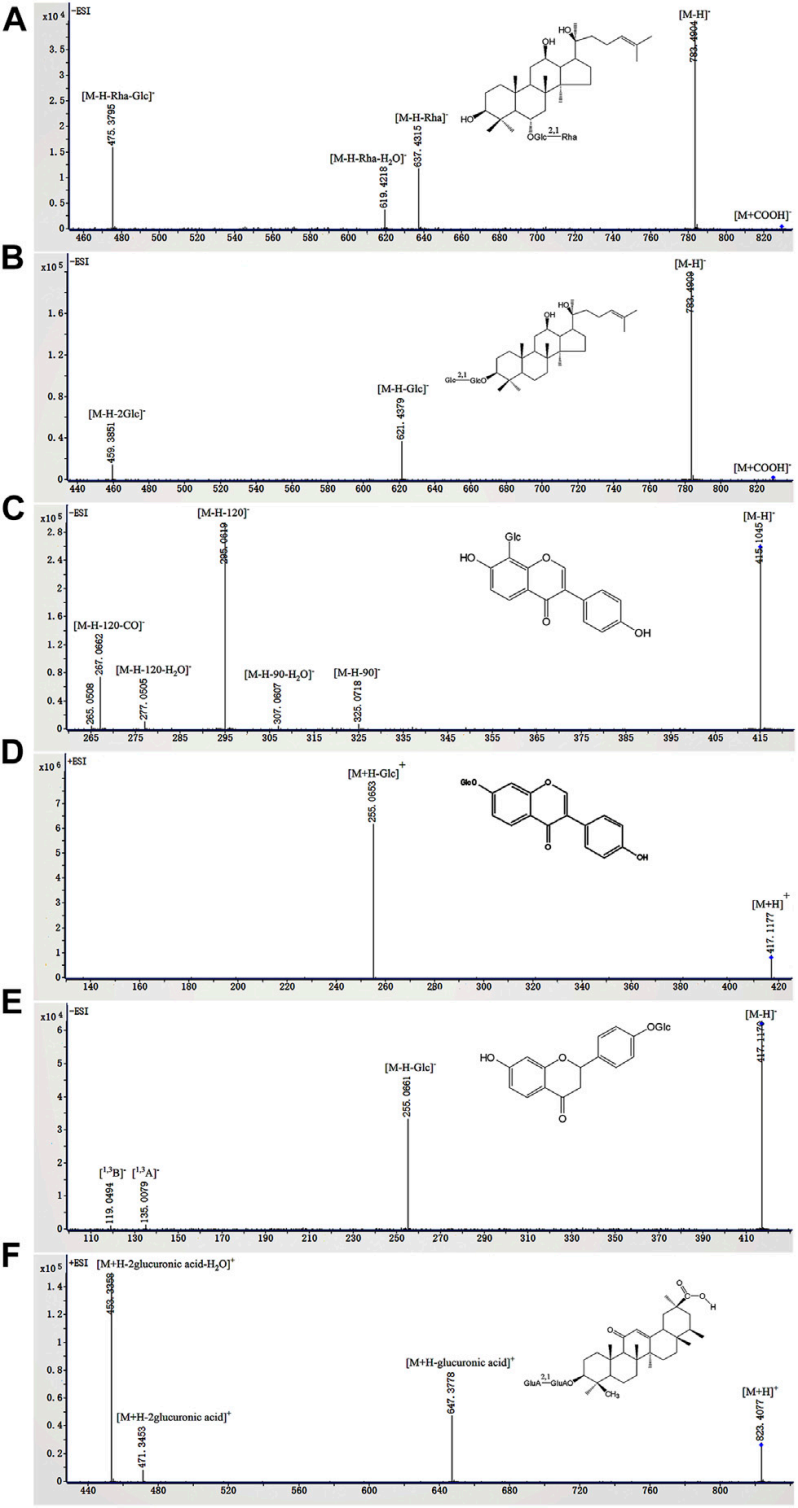
The fragmentation ions of representative compounds in QWBZP-TG. **(A)** ginsenoside Rg_2_, **(B)** ginsenoside Rg_3_, **(C)** puerarin, **(D)** daidzin, **(E)** liquiritin, and **(F)** glycyrrhizic acid.

According to previous reports (Peng et al., 2011; Miao et al., 2013; Yan et al., 2013; Zhou et al., 2013; Hao et al., 2014; Li et al., 2015; Wang H.-P. et al., 2016; Zhao et al., 2017; Guo et al., 2020; Shi et al., 2020; Xie L. X. et al., 2021; Chang et al., 2021; Fu et al., 2021), the identified glycosides were mainly derived from *Panax ginseng*, *Pueraria lobata*, *Glycyrrhiza uralensis*, and *Pogostemon cablin*. These compounds can be classified into eight classes including triterpenoid saponins (compounds **1**–**27**), isoflavone glycosides (compounds **28**–**50**), flavone glycosides (compound **51**), flavanone glycosides (compounds **52**, **53**), chalcone glycosides (compounds **54**, **55**), phenylethanoid glycoside (compounds **56**–**58**), lignan glycoside (compound **59**), and aglycone (compounds **60**–**68**). Among them, compounds **3** (ginsenoside Rg_1_), **4** (ginsenoside Re), **5** (ginsenoside Ro), **32** (puerarin), **35** (daidzin), **54** (liquiritin), and **61** (liquiritin apioside) were confirmed by comparison with the reference standards. The BPC and fragmentation ions of the reference standards are presented in Supplementary Figure S1 and Supplementary Figure S2, respectively. The representative compounds in QWBZP-TG are presented in Figure 2.

The abundance of the components was measured by peak area percentage. Puerarin (7.25%), 3′-methoxypuerarin (5.48%), 3′-hydroxypuerarin (3.79%), 6″-*O*-xylosylpuerarin (3.62%), and daidzin (2.31%) were the five most abundant compounds in QWBZP-TG, owing to the large amount of *Pueraria lobata* in the formula.

### Identification of the *in vitro* Metabolites of QWBZP-TG

**FIGURE 3:**
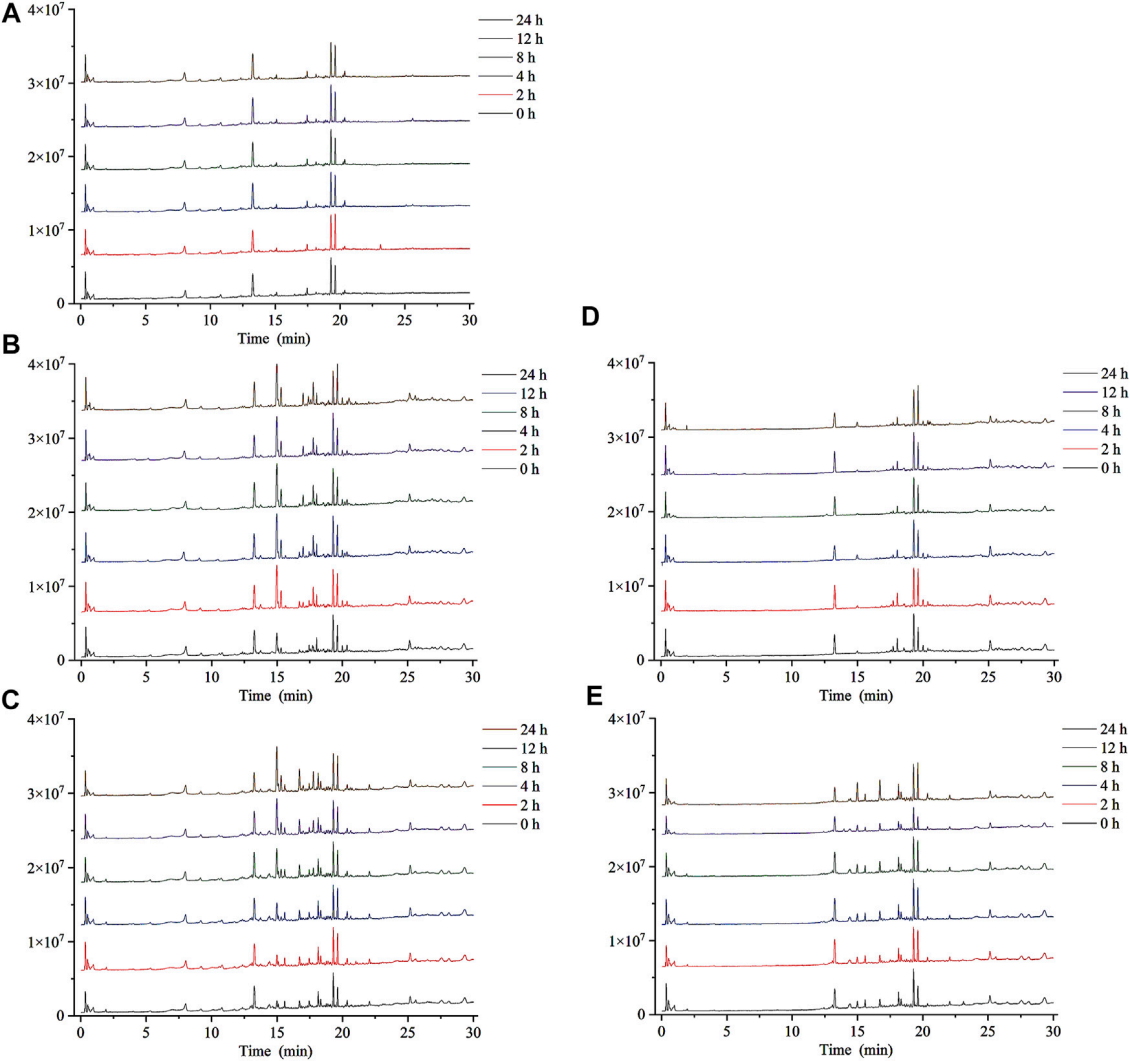
The BPCs of metabolic samples. **(A)** Y, **(B)** NG, **(C)** MG, **(D)** N, and **(E)** M.

**FIGURE 4:**
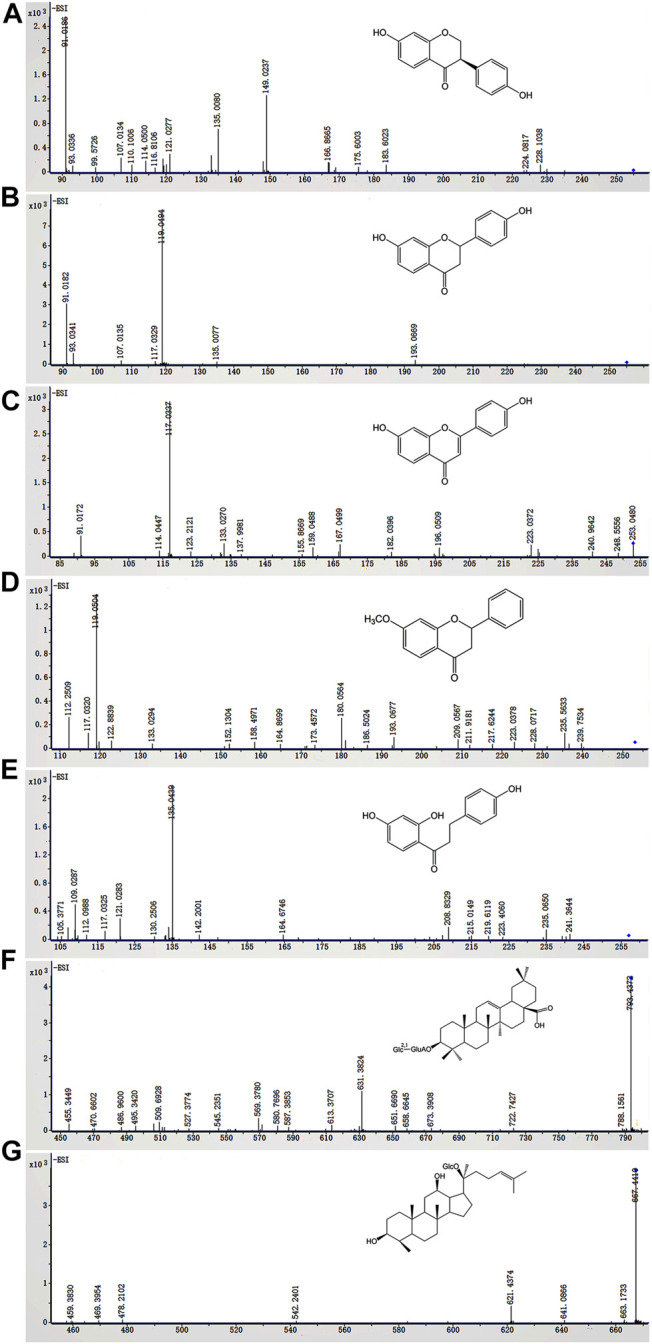
The *in vitro* metabolites of QWBZP-TG **(A)** dihydrodaidzein, **(B)** liquiritigenin, **(C)** dehydroliquiritigenin, **(D)** 4′-dehydroxyliquiritigenin-7-methyl ether, **(E)** dihydroisoliquiritigenin, **(F)** zingibroside R1, and **(G)** compound **(K)**

In this study, ESI^−^ was more sensitive and suitable for QWBZP-TG analysis than ESI^+^. Therefore, ESI^−^ was selected for the identification of QWBZP-TG metabolites. All components except for licorice saponin G_2_, licorice saponin A_3_, ginsenoside Re_1/2/3_, ginsenoside Rb_2_/Rb_3_, ginsenoside Rg_9_, and ginsenoside Rs_4_ were detected in the metabolic samples of Y_0_, NG_0_, and MG_0_. The BPCs of the different groups at different times are shown in Figure 3. Seven potential metabolites were identified in this study. The MS data of the metabolites are summarized in Table 3 and the product ion spectra of the metabolites are shown in Figure 4.

**TABLE 3:** Identification of *in vitro* metabolites of QWBZP-TG.

No.	*t*_R_/min	Identification	Formula	[M-H]^-^/[M + COOH]^-^			Fragment Ions
				Detected	Expected	Error/ppm	
M1	14.876	dihydrodaidzein^a^	C_15_H_12_O_4_	255.0661	255.0663	0.71	149.0237, 135.0080, 121.0277, 91.0186
M2	15.074	liquiritigenin^a^	C_15_H_12_O_4_	255.0660	255.0663	1.10	149.0246, 119.0494, 117.0329, 107.0135, 91.0182
M3	14.744	dehydroliquiritigenin	C_15_H_10_O_4_	253.0504	253.0506	0.91	225.0504, 197.0598, 133.0270, 117.0337, 91.0172
M4	14.581	4′-dehydroxyliquiritigenin-7-methyl ether	C_16_H_14_O_3_	253.0863	253.0870	2.83	239.7534, 133.0294, 119.0504, 117.0320
M5	15.181	dihydroisoliquiritigenin	C_15_H_14_O_4_	257.0817	257.0819	0.90	135.0439, 121.0283, 117.0325, 107.0496
M6	17.867	zingibroside R1	C_42_H_66_O_14_	793.4385	793.4380	-0.65	631.3824, 587.3853, 455.3449
M7	20.505	compound K	C_36_H_62_O_8_	667.4407	667.4427	3.17	621.4374, 459.3830

a Compared with reference standards.

**FIGURE 5:**
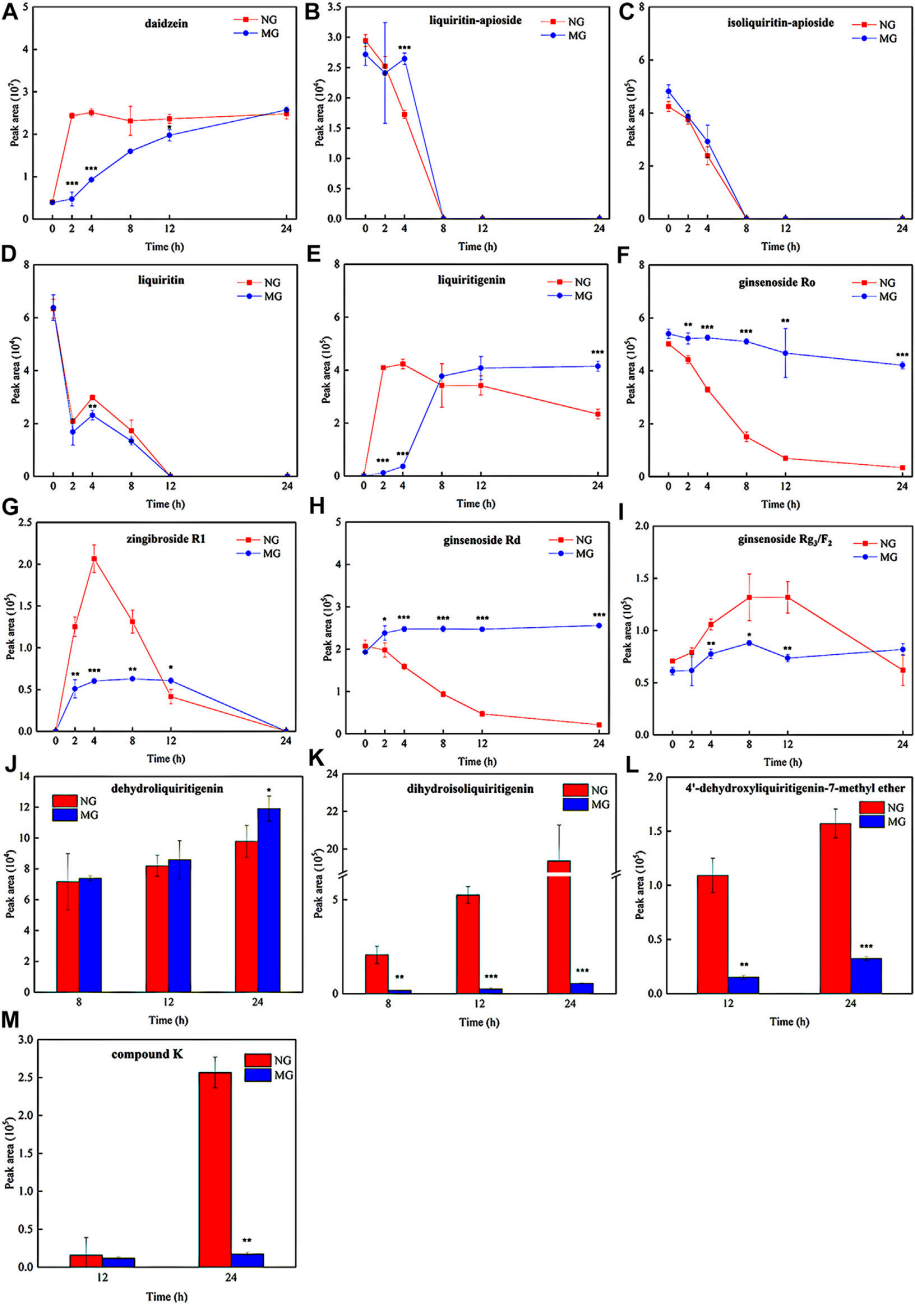
The metabolic ratios of glycosides and corresponding metabolites of normal and diarrheal mice. Each data point is expressed as mean ± SD (*n* = 3). ^*^ *p* < 0.05, ^**^ *p* < 0.01, ^***^ *p* < 0.001.

The characteristic ions produced by the Retro-Diels-Alder (RDA) reaction (*m/z* 149, 135, 133, 119, 117, and 91) were found in **M1**–**M4**, which indicated that they were the metabolites of flavone glycosides. **M1** exhibited an [M-H]^-^ ion at *m/z* 255.0661 (C_15_H_12_O_4_), which was 2 Da (2 H) higher than that of daidzein. Based on fragmentation and reference standard, **M1** was identified as dihydrodaidzein, the reduction product of daidzein (Prasain et al., 2004) (Figure 4A). **M2**–**M4** are metabolites that were transferred from liquiritin and its related compounds. **M2** was eluted at 15.074 min and showed an [M-H]^-^ ion at *m/z* 255.0660 (C_15_H_12_O_4_), which was 162 Da lower than that of liquiritin, indicating the absence of a Glc moiety. The typical ions at *m/z* 135, 119, and 91 produced by RDA cleavage were in accordance with the reference standard. Thus, **M2** was confirmed as liquiritigenin (Figure 4B), a hydrolysis metabolite of liquiritin. The deprotonated ions of **M3** (Figure 4C) and **M5** (Figure 5E) were 2 Da lower and 2 Da higher than that of **M2**, respectively. Prominent ions were formed by the RDA reaction. Based on fragment information and literature data (Li et al., 2018; Zhang et al., 2020; Wu et al., 2021), **M3** and **M5** were suggested to be the dehydrogenation and hydrogenation products of **M2**, respectively. The fragment ion of **M4** at *m/z* 239 (-14 Da) indicated the presence of methyl. According to a previous report (Zhang et al., 2020), **M4** was deduced to be 4′-dehydroxyliquiritigenin-7-methyl ether (Figure 4D).

**M6** and **M7** were metabolites of ginsenosides. **M6** showed a deprotonated ion [M-H]^-^ at *m/z* 793.4385 (C_42_H_66_O_14_), which was 162 Da less than that of the ginsenoside Ro. The fragment ions at *m/z* 631, 587, and 455 were attributed to the successive loss of Glc, CO_2_, and GlcA, respectively (Figure 4F). The fragmentation behavior was in accordance with literature data for zingibroside R1 (Wang J. et al., 2016). Consequently, **M6** was identified as zingibroside R1. **M7** gave [M + COOH]^-^ ions at *m/z* 667.4407. Moreover, the [M-H]^-^ ion at *m/z* 621.4347 and characteristic ions of the protopanaxadiol (PPD)-type aglycone at *m/z* 459 were observed (Figure 4G). Based on the abovementioned information, **M7** was identified as compound K, which is a hydrolysis metabolite of the ginsenoside Rg_3_ or F_2_ (Wang et al., 2014).

### Characteristics of QWBZP-TG Metabolized by the GM of Normal and Diarrheal Mice

To compare the differences in QWBZP-TG metabolized by the GM of normal and diarrheal mice, all metabolic samples from different groups and times were analyzed. The relative content of the components was evaluated based on the peak area. The results showed that the metabolic profiles of QWBZP-TG between the NG and MG groups were similar. In total, most of the components did not change significantly over time. Deglycosylation, dehydrogenation, hydrogenation, and methylation reactions were observed and showed stage characteristics. Among them, deglycosylation was the primary reaction during the whole process of *in vitro* metabolism, while dehydrogenation, hydrogenation, and methylation occurred after 4 h. Although most of the components did not change significantly in the two groups, the metabolic rate of several components, including liquiritin, liquiritin apioside, isoliquiritin apioside, ginsenoside Ro, ginsenoside Rd, and ginsenoside Rg_3_/F_2_, was different between NG and MG groups (Figure 5). No significant differences were observed among the Y_0_, NG_0_, and MG_0_ samples. Subsequently, the metabolic differences increased with time.

The daidzein (the aglycone of puerarin and daidzin) content increased with time in both groups (Figure 5A). Daidzein increased sharply at 0–2 h in the NG group and then stabilized, while it increased continuously from 0–24 h in the MG group. The daidzein contents in MG_2_ (*p* = 0.000), MG_4_ (*p* = 0.000), and MG_12_ (*p* = 0.018) were significantly lower than those in NG_2_, NG_4_, and NG_12_. Surprisingly, no significant differences were observed between NG_24_ and MG_24_. The results indicated that the GM of normal mice exhibited strong deglycosylation capability at 0–2 h, whereas deglycosylation occurred persistently in the GM of diarrheal mice at 0–24 h.

Liquiritin apioside, isoliquiritin apioside, and liquiritin exhibited similar metabolic behaviors. As shown in Figures 5B,C, liquiritin apioside and isoliquiritin apioside decreased from 0–8 h in both NG and MG groups and were not detected in the samples after 8 h, suggesting that the two components were completely degraded by the GM within 8 h. Similarly, liquiritin decreased significantly during 0–2 h and then continuously during 4–12 h in both groups, and it was not detected after 12 h (Figure 5D). Liquiritigenin was found in the two groups from 2–24 h (Figure 5E). The liquiritigenin contents in NG_2_ and NG_4_ were 35.7- and 11.7- fold greater than those in MG_2_ (*p* = 0.000) and MG_4_ (*p* = 0.001), respectively. However, the liquiritigenin content in MG grew sharply at 4–8 h and exceeded that in NG. It was evident that the deglycosylation ability of diarrheal mice GM recovered gradually, which may due to the proliferation of bacteria which is responsible for the deglycosylation process.

Dehydrogenation and hydrogenation of liquiritigenin was observed in the two groups at 8 h, indicating that oxidation and reduction reactions occurred after deglycosylation. Importantly, the dehydrogenation of liquiritigenin in the GM of diarrheal mice was more powerful than that in the GM of normal mice (*P*_24_ = 0.050) (Figure 5J), while hydrogenation of diarrheal mice was weaker than that in normal mice (*P*_8_ = 0.019; *P*_12_ = 0.000; *P*_24_ = 0.000) (Figure 5K). Interestingly, dihydrodaidzein was detected only in NG_24_, which supports the aforementioned conclusion. 4′-dehydroxyliquiritigenin-7-methyl ether was detected in the two groups at 12 h (Figure 5L), and its content was higher in NG than that in MG (*P*_12_ = 0.001; *P*_24_ = 0.000). This implies that methylation occurred later than the oxidation and reduction reactions in this study.

PPD-type and oleanane (OLE)-type ginsenosides were more easily metabolized than protopanaxatriol (PPT)-type ginsenosides in both NG and MG groups. The ginsenoside Ro content decreased over time in both groups (Figure 5F) and was transferred to zingibroside R1. Zingibroside R1 surged from 0–4 h and then dropped from 4–24 h in NG (Figure 5G), implying that zingibroside R1 was further converted to other metabolites. However, the reduction in zingibroside R1 began at 12 h in the MG group. The content difference of zingibroside R1 in the two groups decreased gradually. The metabolic ratios of ginsenosides Rd and Rg_3_/F_2_ in the two groups are shown in Figures 5H,I. Compound K, a metabolite derived from ginsenoside Rg_3_/F_2_, was detected in both groups at 12 h (Figures 5M). The content of compound K in NG_24_ was higher than that in MG_24_ (*p* = 0.002).

## Discussion

Deglycosylation is the main type of glycoside hydrolysis that occurs in the intestine (Xu et al., 2017). The GM secretes abundant glycoside hydrolases, such as *β*- *d* -glucosidase, *β*- *d* -glucuronidase, *β*-xylosidase, and *α*- *l* -rhamnosidase, which contribute to the stepwise cleavage of sugar moieties (Xu et al., 2017). Glycosyl and glucuronosyl moieties are the representative groups of glycosides in QWBZP-TG. In this study, GM metabolized QWBZP-TG *via* a multi-step reaction. An abundance of deglycosylation products was detected in the biotransformation samples, while metabolites produced by hydrogenation and dehydrogenation were found at 8 h and methylation metabolites at 12 h. The rate of deglycosylation, hydrogenation, and methylation were faster in the GM of normal mice than that in the GM of diarrheal mice, which is in accordance with the report of Ling et al. (2018). Furthermore, dihydrodaidzein was only detected in the fecal incubation solution of normal mice. According to our previous study (Shao et al., 2020), the GM diversity diminished after antibiotic administration. In particular, lower activities of *β*- *d* -glucosidase and *β*- *d* -glucuronidase were observed in diarrheal mice compared to those in normal mice (in press). *Bifidobacteria* and *Lactobacillus*, which possess glycosyl hydrolase, were promoted by QWBZP-TG *in vivo* (Xie G. Z. et al., 2021). Thus, the decrease in GM abundance and enzyme activity may result in slower QWBZP-TG metabolism in diarrheal mice. Although slower deglycosylation was observed in the GM of diarrheal mice, persistent deglycosylation may help diarrheal mice to produce adequate saccharides and aglycones. These two components can improve diarrhea by promoting the proliferation of beneficial bacteria and exerting anti-inflammatory effects, respectively. In turn, the growth of glycoside-hydrolyzing bacteria accelerated the deglycosylation reaction. Actually, we also noted that the content differences of daidzein and liquiritigenin between the two groups narrowed from 8 h. Additionally, the peak areas of the two aglycones at 24 h in the GM of diarrheal mice were larger than that of normal samples. This result confirms the abovementioned hypothesis. As a result, glycoside-bacteria interaction may be a key mechanism for QWBZP in diarrhea therapy.

The selective/preferential metabolism of the GM of a system containing multiple ingredients is an issue of concern. It has been reported that many members of the GM were predisposed to metabolize monosaccharides, disaccharides, and/or oligosaccharides (Turroni et al., 2018). In addition, the GM exhibits structural specificity for drug metabolism (Zimmermann et al., 2019b). For example, drugs that contain ester or amide groups can be specifically metabolized by *Bacteroidetes*, and compounds with nitro or azo groups can be metabolized by most bacteria, except Proteobacteria (Zimmermann et al., 2019b). Flavonoids with hydroxyl groups at the 5-, 7-, and 4′-positions are degraded more quickly by the GM (Ling et al., 2021). In this study, flavone glycosides and triterpenoid saponins were the two main types of glycosides detected in QWBZP-TG. Importantly, GM metabolizes liquiritin apioside (flavanones glycoside), isoliquiritin apioside (chalcones glycoside), and liquiritin (flavanones glycoside) more effectively than other flavone glycosides under the same conditions, in agreement with earlier studies (Huang et al., 2012). All of them had a glucose unit at the 4′ position of the B ring. Thus, the small number of sugar units and steric hindrance can explain why they are easier to hydrolyze. According to Li et al. (2017), the metabolic efficiency of PPD-type ginsenosides was higher than that of PPT-type ginsenosides, and the superior deglycosylation sequence was *C*-3 > *C*-20 > *C*-6, which was also supported by our study. Interestingly, ginsenoside Ro was also relatively easy to metabolize by normal mice GM, which was consistent with a previous report (Wan et al., 2013). Compared to other ginsenosides, the glucose group of ginsenoside Ro is dehydrated with the carboxyl group at *C*-28 to form an esteroside bond, leading to easier hydrolysis. Ginsenoside Ro was transformed into zingibroside R1 after removing the glucose group of *C*-28.

QWBZP-TG is a complex system that includes glycosides and aglycones, which are mutually converted into each other during intestinal metabolism. Consequently, many compounds are both prototypes and metabolites. Thus, it is challenging to clarify the metabolic pathways and metabolites of QWBZP-TG. Taking puerarin, daidzin, and daidzein as examples, daidzein is the hydrolysis metabolite of puerarin and daidzin, all of which were detected in the QWBZP-TG. The amount of daidzein increased with time in GM biotransformation samples. However, the amounts of puerarin and daidzin did not show an obvious decline. Puerarin is a *C*-glycoside that is difficult to hydrolyze. 6″-*O*-acetyl daidzin and daidzein-4′,7-*O*-glucoside were metabolized to daidzin and daidzein, respectively, which contributed to the changes in the daidzin and daidzein contents. On the other hand, stepwise deglycosylation is the main metabolic behavior of ginsenosides in the intestine. Hence, it is difficult to obtain clear metabolic rules for systems containing multiple ginsenosides. In addition, the number GM members that can be aerobically cultured *in vitro* is limited; therefore, the *in vitro* GM metabolism can only reflect a part of the intestinal metabolism. Further studies on the *in vivo* GM metabolism are required.

## Conclusion

This study aimed to investigate the *in vitro* metabolic profiles and differences of multi-glycosides in the GM of normal and diarrheal mice. The results suggest that the GM metabolizes multi-glycosides through several reactions, among which deglycosylation is the primary and dominant reaction. Moreover, the deglycosylation rate in the GM of normal mice was faster than that in the GM of diarrheal mice. However, the deglycosylation capability of diarrheal mice gradually recovered. The abundant deglycosylation metabolites may help regulate the GM and further improve diarrhea. In the multi-glycoside system (from QWBZP), liquiritin apioside, isoliquiritin apioside, liquiritin, and PPD-type and OLE-type ginsenoside were degraded more quickly by GM than other glycosides.

## Data Availability

The original contributions presented in the study are included in the article/Supplementary Material. Further inquiries can be directed to the corresponding author.
