# Supplementary figures and images for: In Vitro Biotransformation of Total Glycosides in Qiwei Baizhu Powder by the Gut Microbiota of Normal and Diarrheal Mice: Novel Insight Into the Biotransformation of Multi-Glycosides by the Gut Microbiota
Source: Front Chem. 2022 Jun 20;10:907886. doi: 10.3389/fchem.2022.907886 (PMC9251009; doi:10.3389/fchem.2022.907886)

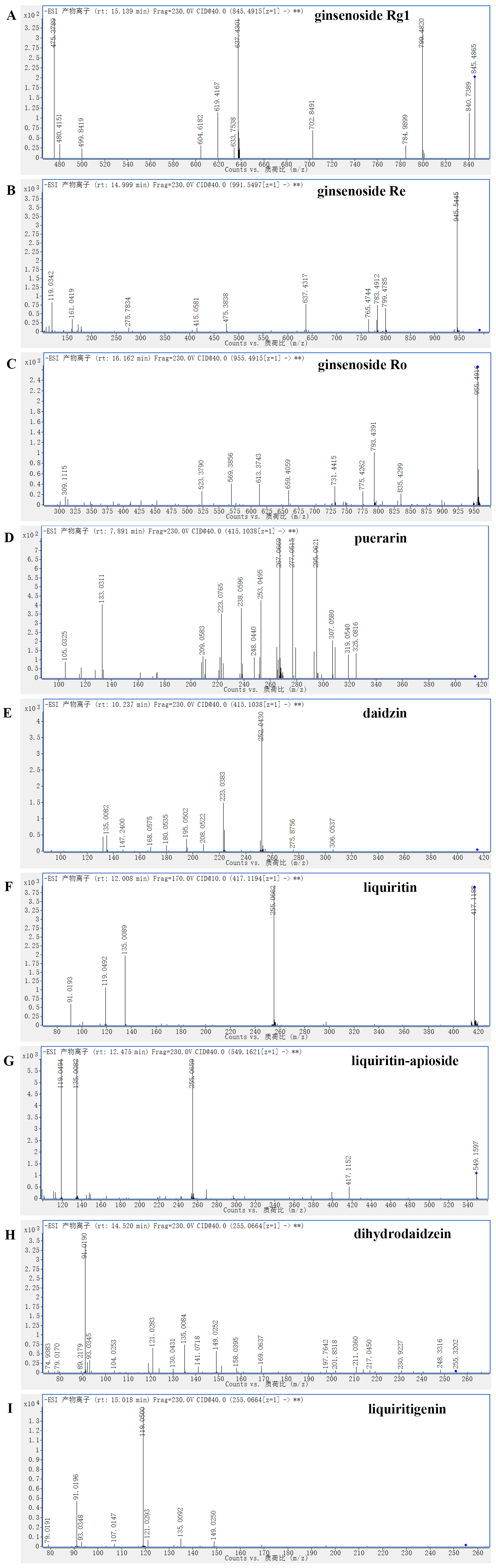

Supplement: Supplementary file 1 [file Image2.TIF]

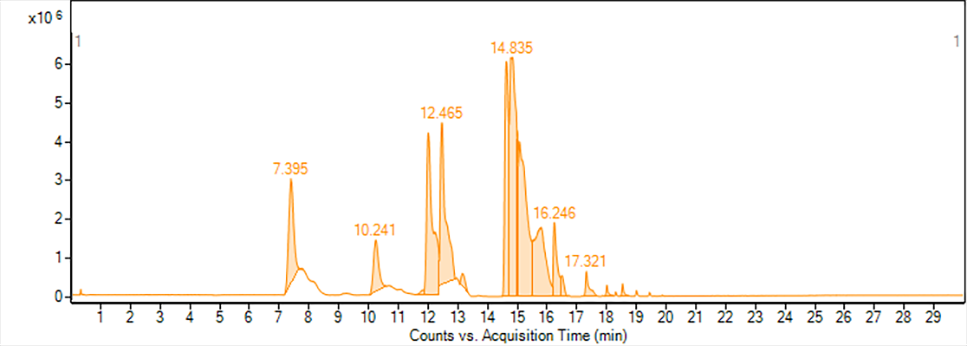

Supplement: Supplementary file 2 [file Image1.TIF]
